# Supplementary figures and images for: MCount: An automated colony counting tool for high-throughput microbiology
Source: PLoS One. 2025 Mar 19;20(3):e0311242. doi: 10.1371/journal.pone.0311242 (PMC11957731; doi:10.1371/journal.pone.0311242)

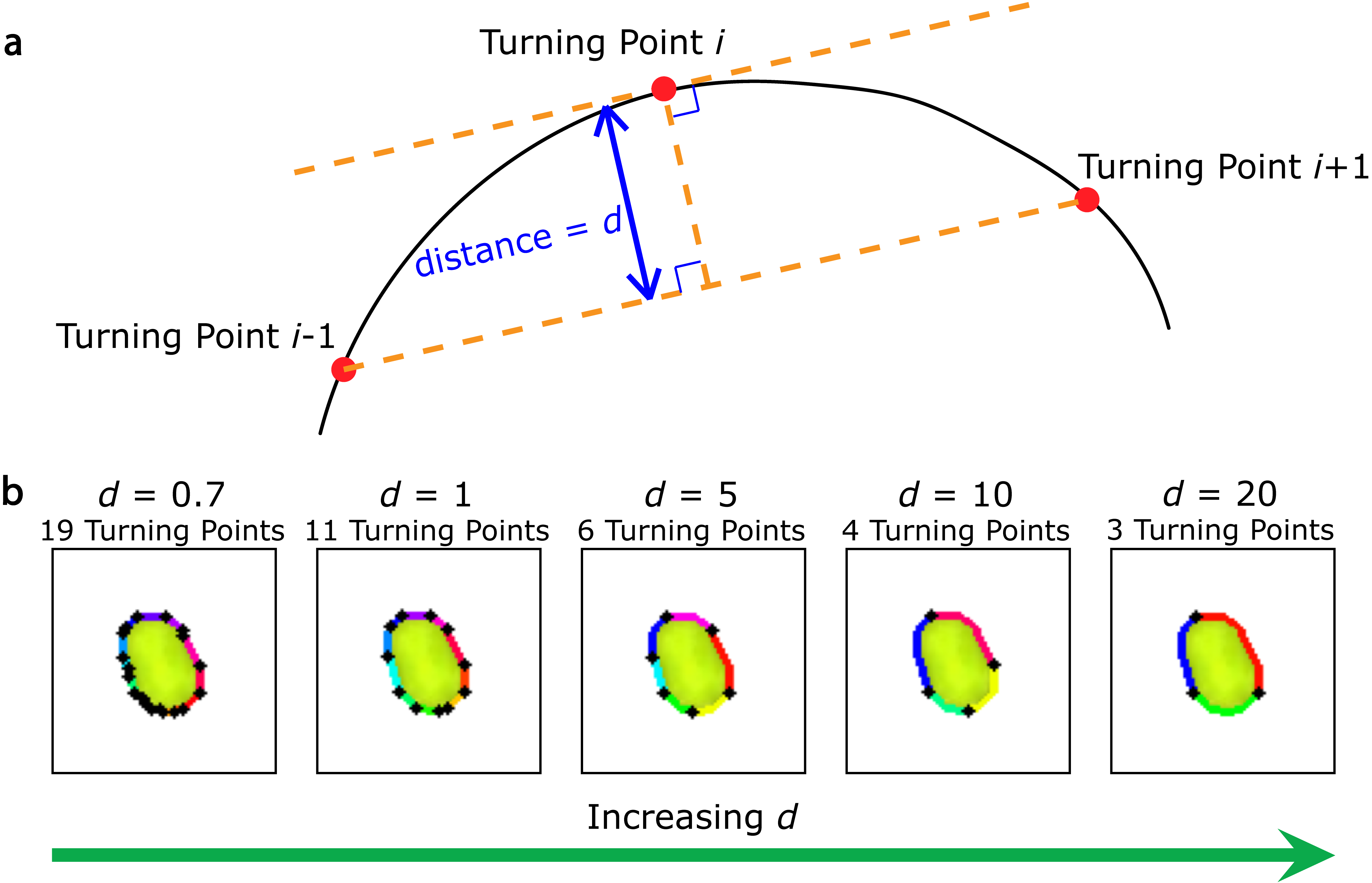

Supplement: S1 Fig — (a) For consecutive three turning points Ti−1, Ti, and Ti+1, d represents the distance from Ti to the line Ti−1Ti+1¯. (b) The larger the value of d, the more turning points are generated to represent the contour. The turning points are represented as black dots, while contour segments divided by turning points are shown in different colors. (TIF) [file pone.0311242.s001.tif]

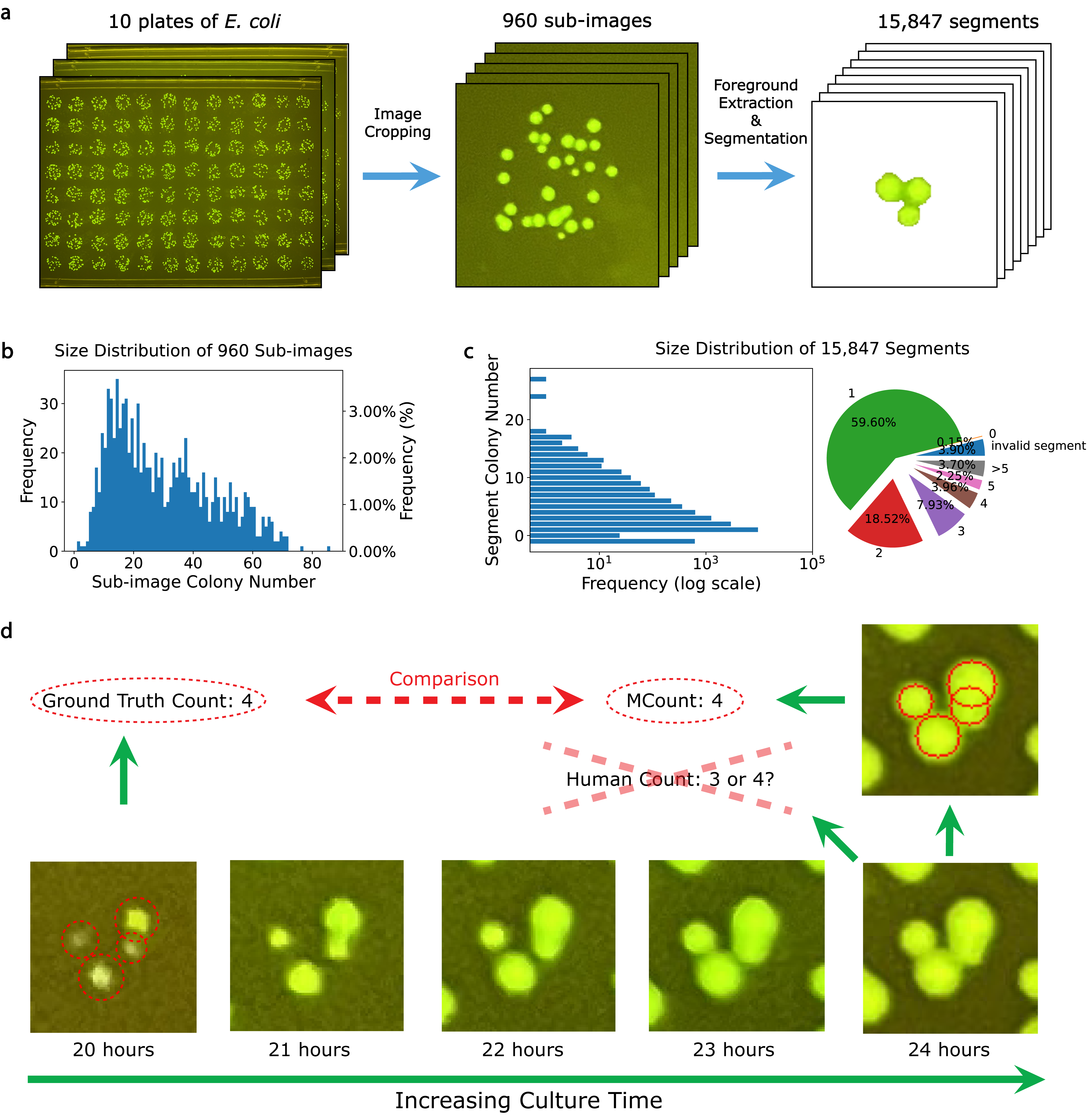

Supplement: S2 Fig — (a) 960 sub-images are obtained by cropping 10 plates of fluorescent E. coli NEB10-beta and further divided into 15,847 segments using foreground extraction and segmentation. (b) The colony number distribution of sub-images shows that most sub-images have 10 ~ 40 colonies. (c) The percentage of single-colony, two-colony, and three-colony segments is 59.6%, 18.52%, and 7.93%, respectively, which takes 86.05% in total. A well-performing algorithm is expected to correctly recognize almost all none and mildly merged colonies. The remaining percentage of segments, merged in a denser manner, requires the algorithm to infer sophisticated shapes. Note that 3.90% of segments are invalid because the merging of colonies is too severe to be labeled correctly, denoted as -1 in the left figure. (d) All segments are labeled according to their shape in a photograph taken about 4 hours ago to ensure labeling accuracy, including segments that are hard for humans to label. (TIF) [file pone.0311242.s002.tif]

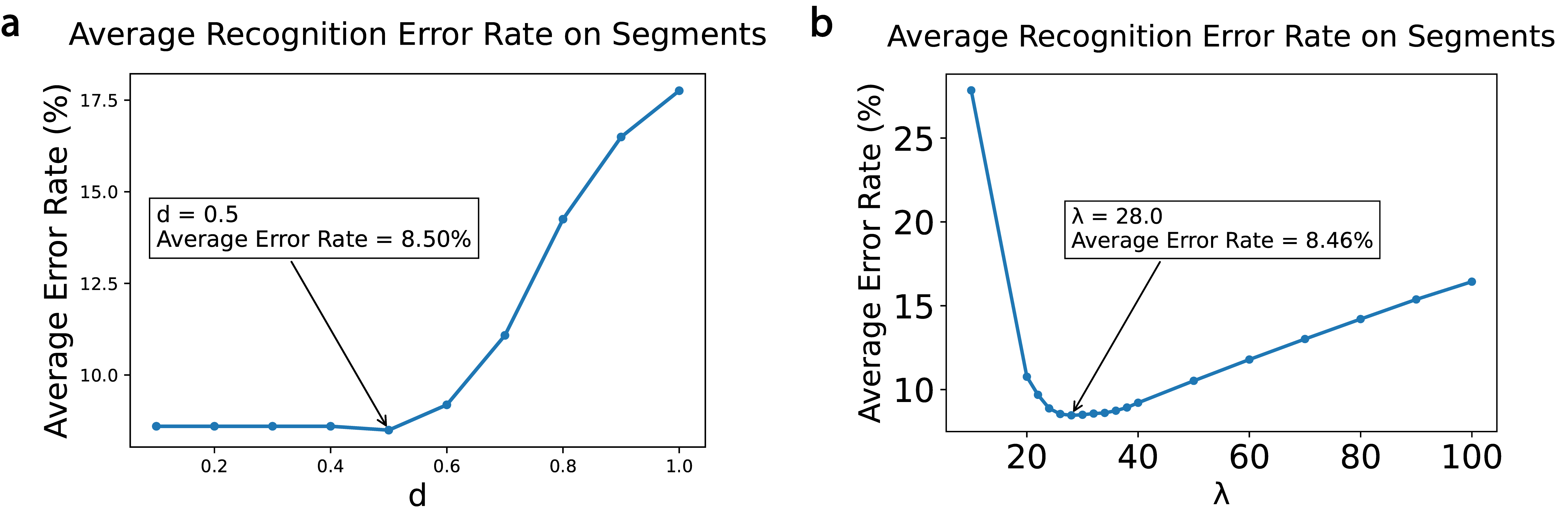

Supplement: S3 Fig — The optimal values of d and λ are determined using a different definition of recognition error. (a) The average error rate on the segment dataset is plotted against d when λ=26. The minimum average error rate of 8.50% is achieved at d=0.5. (b) The average error rate on the segment dataset is plotted against λ when d=0.5. The minimum average error rate of 8.46% is achieved at λ=28. An error of each segment is defined as 1MCount≠Label and the error was averaged across all segments, denoting the average error rate. (TIF) [file pone.0311242.s003.tif]

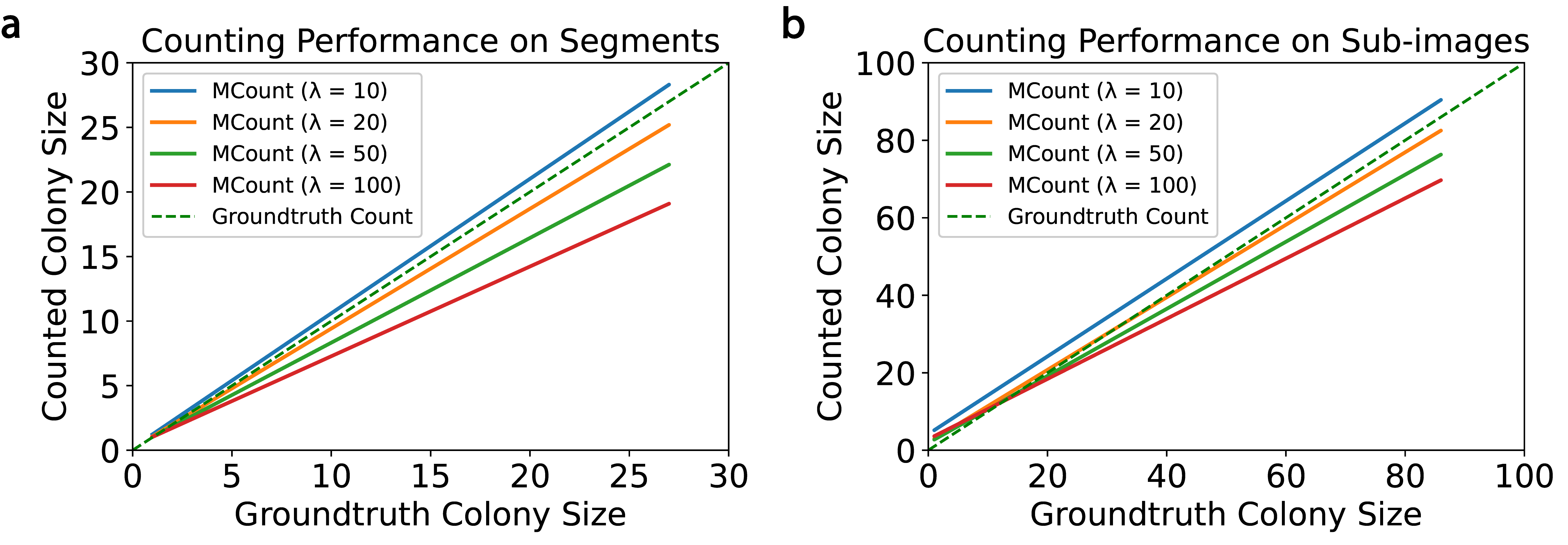

Supplement: S4 Fig — Increasing λ results in monotonously less counting leading to underestimation of colony number. The plot shows the MCount counting result with different λ values versus ground truth label on (a) 15,847 colony segments and (b) 960 sub-images, respectively. The green dashed line represents the regression line for a perfect colony estimator that always gives the result as the label, while the colored line represents the regression line for MCount. By tuning λ, it is possible to address the overestimation/underestimation issue. Increasing λ results in a monotonous decrease in the number of colonies counted, leading to an underestimation of colony number. (TIF) [file pone.0311242.s004.tif]
