# Supplementary material for: MCount: An automated colony counting tool for high-throughput microbiology
Source: PLoS One. 2025 Mar 19;20(3):e0311242. doi: 10.1371/journal.pone.0311242 (PMC11957731; doi:10.1371/journal.pone.0311242)
Supplement: S1 Table — Mean and variance of colony number, optimal hyperparameter values, and the corresponding recognition error rate for each sub-dataset. The results demonstrate minimal variation in hyperparameters across sub-datasets and consistently lower error rates compared to the global settings (3.99%). (DOCX) [file pone.0311242.s005.docx]

| **Dataset** | **Colony Number** | | **Optimized Hyperparameters** | | **Recognition Error Rate, Mean (%)** |
| --- | --- | --- | --- | --- | --- |
|  | **Mean** | **Variance** | $\boldsymbol{d}$ | $\boldsymbol{\lambda}$ |  |
| 1 | 58.79 | 85.24 | 0.1~0.4 | 10 | 3.65 |
| 2 | 20.64 | 53.21 | 0.1~0.4 | 28 | 1.51 |
| 3 | 48.01 | 42.07 | 0.5 | 20 | 3.43 |
| 4 | 15.51 | 28.59 | 0.6 | 36 | 1.58 |
| 5 | 9.38 | 9.89 | 0.7 | 60 | 1.36 |
| 6 | 34.60 | 43.73 | 0.1~0.4 | 24 | 3.09 |
| 7 | 22.33 | 21.97 | 0.6 | 32 | 2.11 |
| 8 | 56.69 | 122.76 | 0.1~0.4 | 18 | 3.99 |
| 9 | 38.28 | 57.64 | 0.1~0.4 | 26 | 2.67 |
| 10 | 15.19 | 23.26 | 0.6 | 50 | 1.56 |
