# Supplementary material for: MCount: An automated colony counting tool for high-throughput microbiology
Source: PLoS One. 2025 Mar 19;20(3):e0311242. doi: 10.1371/journal.pone.0311242 (PMC11957731; doi:10.1371/journal.pone.0311242)
Supplement: S2 Table — Performance of MCount using globally optimized hyperparameters (λ=26 and d=0.5) across 10 sub-datasets. The table presents the average error rates for each sub-dataset when applying the global optimal hyperparameters. The results indicate that MCount maintains robust performance across varied experimental conditions, with error rates significantly lower than those achieved by NICE. (DOCX) [file pone.0311242.s006.docx]

| **Dataset** | **Colony Number** | | **Recognition Error Rate (MCount)** | | **Recognition Error Rate (NICE)** | |
| --- | --- | --- | --- | --- | --- | --- |
|  | **Mean** | **Variance** | **Mean (%)** | **Median (%)** | **Mean (%)** | **Median (%)** |
| 1 | 58.79 | 85.24 | 5.10 | 4.55 | 18.13 | 18.52 |
| 2 | 20.64 | 53.21 | 1.82 | 0 | 11.84 | 10.91 |
| 3 | 48.01 | 42.07 | 4.38 | 3.45 | 19.20 | 17.95 |
| 4 | 15.51 | 28.59 | 3.55 | 0 | 11.95 | 9.52 |
| 5 | 9.38 | 9.89 | 7.87 | 0 | 14.62 | 11.11 |
| 6 | 34.60 | 43.73 | 3.48 | 2.90 | 22.85 | 22.40 |
| 7 | 22.33 | 21.97 | 2.49 | 0.00 | 11.34 | 11.11 |
| 8 | 56.69 | 122.76 | 5.39 | 4.92 | 23.17 | 22.86 |
| 9 | 38.28 | 57.64 | 2.67 | 2.53 | 23.25 | 24.03 |
| 10 | 15.19 | 23.26 | 3.18 | 0 | 9.53 | 9.09 |
