# Supplementary material for: MCount: An automated colony counting tool for high-throughput microbiology
Source: PLoS One. 2025 Mar 19;20(3):e0311242. doi: 10.1371/journal.pone.0311242 (PMC11957731; doi:10.1371/journal.pone.0311242)
Supplement: S3 Table — The optimization solver for MCount is ‘COIN_CMD’ in PuLP, and the solver can be changed to achieve faster processing times at the cost of sacrificing accuracy. (DOCX) [file pone.0311242.s007.docx]

| Algorithms / Software | Total Processing Time (Second) |
| --- | --- |
| **MCount** | 169.8 |
| **NICE** | 15.9 |
| **OpenCFU** | 13.2 |
| **AutoCellSeg** | 79.4 |
