# Supplementary material for: MCount: An automated colony counting tool for high-throughput microbiology
Source: PLoS One. 2025 Mar 19;20(3):e0311242. doi: 10.1371/journal.pone.0311242 (PMC11957731; doi:10.1371/journal.pone.0311242)
Supplement: S4 Table — The mean and variance values were calculated from the 96 colony counts of each dataset. The first column represents the dataset index. The second column represents the mean value of the 96 colony counts, and the third column represents the variance of the 96 colony counts. The 4th and 5th columns show the p-values for Kolmogorov–Smirnov (KS) tests for Normal and Poisson distributions, respectively, under the significant level of 0.05, where no rejection is made for any datasets. The 6th column shows the p-values for Poisson Dispersion tests, and datasets whose null hypotheses are rejected under the significant level of 0.05 are denoted with a star symbol * . (DOCX) [file pone.0311242.s008.docx]

| **Dataset** | **Mean** | **Variance** | **KS test (Normal)** | **KS test (Poisson)** | **Poisson Dispersion test** |
| --- | --- | --- | --- | --- | --- |
| 1 | 58.79 | 85.24 | 0.6604 | 0.6539 | 0.0747 |
| 2 | 20.64 | 53.21 | 0.7431 | 0.3207 | 2.11e-07* |
| 3 | 48.01 | 42.07 | 0.8749 | 0.8492 | 0.6450 |
| 4 | 15.51 | 28.59 | 0.6963 | 0.4516 | 0.0021* |
| 5 | 9.38 | 9.89 | 0.8258 | 0.7202 | 0.4385 |
| 6 | 34.60 | 43.73 | 0.3595 | 0.0523 | 0.0658 |
| 7 | 22.33 | 21.97 | 0.6214 | 0.0947 | 0.5507 |
| 8 | 56.69 | 122.76 | 0.2082 | 0.0974 | 7.95e-06* |
| 9 | 38.28 | 57.64 | 0.8063 | 0.3398 | 0.0087* |
| 10 | 15.19 | 23.26 | 0.3048 | 0.3699 | 0.0017* |
