# Supplementary material for: MCount: An automated colony counting tool for high-throughput microbiology
Source: PLoS One. 2025 Mar 19;20(3):e0311242. doi: 10.1371/journal.pone.0311242 (PMC11957731; doi:10.1371/journal.pone.0311242)
Supplement: S1 File — Discussion, cultivation and preparation on E. coli Dataset used in this study. (DOCX) [file pone.0311242.s009.docx]

**Discussion on *E. coli* Dataset**

In this study, we used GFP fluorescent *E. coli* strain NEB10-beta (New England Biolabs) to create the dataset. We chose to use the GFP fluorescent strain because accurately labeling nonfluorescent plates using the human eye is challenging, even though MCount can accurately recognize nonfluorescent colonies. We also want to address problems (b) and (c) in this section.

In cases where a labeled image of a dataset has too many colonies (more than 150), an algorithm may struggle to accurately identify all colonies even if it provides the same counting number as the label. This can occur due to under-counting issues such as colony merging or over-counting issues like incorrectly identifying image flaws or agar defects as colonies. To address this issue, each sub-image was further decomposed into individual colony segments using foreground extraction, resulting in a dataset of 15,847 segments, as shown in S2 Fig (a-c) shows the colony number distribution of sub-images and segments, respectively, where most sub-images have 10 ~ 60 colonies, and most segments only have few colonies that are less than five. While high accuracy on sub-images does not necessarily guarantee high accuracy on segments, a good performance on segments usually implies an accurately counting on sub-images. Furthermore, as 59.6% segments are single-colony and about 40% segments have more than two colonies, a good algorithm should be capable of accurately recognizing both single colonies and overlapped colonies. Thus, evaluating MCount on segments rather than sub-images is a more reasonable approach.

Problem (c) arises from the difficulty of correctly labeling certain images, such as those with highly overlapped colonies, even for humans. While some datasets simply discard these difficult images, others may take the average of the labeling numbers provided by multiple individuals. However, the former approach leads to a dataset with selection bias, and the latter approach cannot guarantee that the average number is the ground truth. To address this issue, we took pictures of the colonies every hour and labeled every colony segment according to its historical image, as shown in S2 Fig (d). This method enables the evaluation of MCount's ability to infer the number of colonies, even when the task is challenging for humans. Furthermore, this approach allows the evaluation of MCount's performance on images with a higher degree of colony merging. An example is shown in S2 Fig (d), where the ground truth colony count is 4, based on the image taken when the colonies had been growing for 20 hours. Looking at the image taken when the colonies had been growing for 24 hours, it is difficult to determine whether there are 3 or 4 colonies, but MCount can correctly recognize the count as 4.

**Cultivation and preparation of *E. coli* dataset**

The first step involved transforming *E. coli* NEB10-beta with a GFP fluorescent plasmid via electroporation. A glycerol stock was used to inoculate 5 mL of LB medium in a 14-mL culture tube, which was incubated overnight at 37˚C and 250 rpm. The following morning, 800 μL of the cell culture was transferred to 80 mL of LB medium and grown at 37˚C and 250 rpm until reaching the exponential phase, which was determined by measuring the OD600 using a UV spectrophotometer (UV-1800, Shimadzu). The cell culture was then centrifuged at 3500 rpm at 4˚C (F0650 rotor, Allegra 64R Benchtop Centrifuge, Beckman Coulter), and the supernatant was discarded. The cell pellets were washed with 2 mL of pre-chilled 10% glycerol at 4 °C, and the process was repeated after two more centrifugations at 8000 rpm at 4˚C (F1202 rotor, Allegra 64R Benchtop Centrifuge). After the final centrifugation, the cell pellets were suspended in 10% glycerol, and the volume was adjusted so that the final cell concentration was about OD600 of 10. Ampicillin resistance and GFP encoding DNA plasmids (Parts Registry K176011) were mixed with the cell sample to obtain a final concentration of 0.1 ng μL^−1^. The sample was loaded into 2 mm VWR electroporation cuvettes, and electroporated using a MicroPulser™ (Bio-Rad) at 2.5 kV with a 6 ms time constant. The electroporated sample was immediately suspended in 900 μL of pre-warmed LB medium at 37˚C and 250 rpm for one hour. Finally, 100 μL of the cell suspension was pipetted onto LB agar plates containing ampicillin (50 μg mL^−1^), which were then incubated overnight at 37˚C and 250 rpm.

In the next step, the GFP fluorescent *E. coli* was plated in a 96-well format. An individual colony was picked from the plate made in the previous step and inoculated in 5 mL of LB medium in a 14-mL culture tube. The culture was incubated overnight at 37˚C and 250 rpm, and 200 μL of the resulting cell culture was transferred to 20 mL of LB medium and grown at 37˚C and 250 rpm until the OD600 reached 0.5. The cell sample was then serially diluted using LB medium to obtain a dilution of about 1×10^7^~ 8×10^7^. A liquid handling robot (Perkin Elmer JANUS G3 BioTx Pro Plus) was used to dispense 5 μL of the sample into each well of an agar plate in an 8×12 format. The dilution ratio could be adjusted to obtain different colony plating densities. The plate was then incubated at 37˚C for 12 hours. Photos were taken once an hour using an iPhone 12 Pro until the colonies were overgrown, which was about 25 hours.
