# Supplementary material for: MCount: An automated colony counting tool for high-throughput microbiology
Source: PLoS One. 2025 Mar 19;20(3):e0311242. doi: 10.1371/journal.pone.0311242 (PMC11957731; doi:10.1371/journal.pone.0311242)
Supplement: S2 File — (DOCX) [file pone.0311242.s010.docx]

**Discussion on the distribution of colony numbers**

Although it is theoretically predicted that colony numbers should follow a Poisson distribution, the distribution may deviate from Poisson and become closer to a Normal distribution due to noise induced by liquid handling operations. We illustrate this phenomenon using our benchmark data.

Our benchmark consists of ten *E. coli* plates, and we investigated the distribution of all ten plates using hypothesis testing. We used the Kolmogorov–Smirnov (KS) test to test the null hypothesis ($H_{0}$) that the distribution of 96 colony numbers follows a Poisson or Normal distribution with a significant level of 0.05. The p-values of the KS test (Table S2) show that we cannot reject the null hypothesis that the distribution follows either a Poisson or Normal distribution for any of the datasets. However, we cannot conclude that they follow Poisson or Normal distribution, either. As the p-values of KS test (Poisson) are smaller than those of KS test (Normal) for almost all datasets, we conclude that our benchmark is closer to a Normal distribution than a Poisson distribution.

We also conducted Poisson Dispersion tests (Table S4) and found that some of the datasets still have the equidispersion property, despite being closer to a Normal distribution.
