# Supplementary material for: MCount: An automated colony counting tool for high-throughput microbiology
Source: PLoS One. 2025 Mar 19;20(3):e0311242. doi: 10.1371/journal.pone.0311242 (PMC11957731; doi:10.1371/journal.pone.0311242)
Supplement: Attachment — Submitted filename: Response to Reviewers.docx [file pone.0311242.s012.docx]

**Response to Editor**

**[Comment]**

Thank you for uploading your study's underlying data set. Unfortunately, the repository you have noted in your Data Availability statement does not qualify as an acceptable data repository according to PLOS's standards.
At this time, please upload the minimal data set necessary to replicate your study's findings to a stable, public repository (such as figshare or Dryad) and provide us with the relevant URLs, DOIs, or accession numbers that may be used to access these data. For a list of recommended repositories and additional information on PLOS standards for data deposition, please see <https://journals.plos.org/plosone/s/recommended-repositories>.

**[Response]**

We appreciate the editor’s time reviewing our manuscript and Data Availability statement. We accept the suggestion and have uploaded the data set to Dryad. The relevant URL or DOI to access the data set is as follows: <https://doi.org/10.5061/dryad.2280gb62f> (forthcoming as of September 5, 2024; for temporary access in the meantime, please access via <https://datadryad.org/stash/share/xh0Ek1hMtE-lihJ5wB590g-rnLcfL6G0irdWlaVxjhE>). We have added the URL to the section “Data Availability” as follows:

“All methods implemented and data used are publicly available on Dryad (<https://doi.org/10.5061/dryad.2280gb62>).”

**Response to Reviewer 1**

**[Comment 1-1]**

In the introduction, the authors discuss several existing tools for colony counting (NICE, AutoCellSeg, OpenCFU), but in the results section they only compare the performance of MCount to NICE. Since the authors went through the effort of creating a ground-truth dataset, it would be nice to see also the performance of the other methods on this dataset.

**[Response 1-1]**

We appreciate the reviewer’s valuable comment. Indeed, we previously did not compare the results of MCount to AutoCellSeg and OpenCFU and instead outlined their limitations as described in Introduction and in Figure 1. Following reviewer’s comment, we have performed colony counting using AutoCellSeg and OpenCFU and have now added their results to Figure 4 for comparison as follows:

**Fig 4. Comparison of MCount (**$\boldsymbol{\lambda=}\mathbf{26}$ **and** $\boldsymbol{d=}\mathbf{0.5}$**) and NICE performance in recognizing colony segments and sub-images. (a)** Bubble plot of MCount counting result versus ground truth label on 15,847 colony segments. The size of each bubble at location (x,y) is proportional to the number of segments that have x merged colonies but are recognized as y colonies by MCount. The blue line represents the regression line for MCount, while the green dashed line represents the regression line for a perfect algorithm that always gives the result as the label. MCount exhibits slight underestimation as the blue line is lower than the dashed green line. **(b)** Rank error curve of error rate values for 15,847 segments. MCount recognizes 91.45% of segments with zero error rate. **(c)** Bubble plot of Mcount, NICE, AutoCellSeg, and OpenCFU counting results versus ground truth count label on 960 colony sub-images. The blue line is much closer to the dashed green line compared to the orange line, indicating that MCount is more accurate in recognizing sub-images. **(d)** Comparison of MCount, NICE, AutoCellSeg, and OpenCFU performance in underestimation or overestimation of colony numbers. MCount has fewer underestimated results and less severe underestimation and overestimation compared to NICE. **(e)** Boxplot of error rate on 960 sub-images for MCount and NICE. MCount has a much lower average error rate and a smaller interquartile range, indicating more consistent performance.

As expected, we see that MCount has shown the lowest error rate compared to other methods such as NICE, AutoCellSeg, and OpenCFU. We have added our motivations and observations of the comparison study as follows (lines 305-309 of main text):

“Consequently, we selected the most popular and widely used tools — NICE (9), OpenCFU (10), AutoCellSeg (11) — for comparison.

Fig 4c is a bubble plot showing all algorithms versus the ground-truth label on sub-images. All algorithms have relatively uniform distributions indicated by the uniform bubble sizes. While MCount only slightly underestimates the number of colonies, which can be addressed by tuning the $\lambda$ value as shown in S4 Fig (b), NICE, AutoCellSeg, and OpenCFU largely underestimates the number of colonies. Since AutoCellSeg tends to count every continuous region as a single colony regardless of its shape, it has the most severe underestimation issue; as OpenCFU struggles with low-resolution images, its fitted line cannot even penetrate the original point (x,y)=(0,0). Fig 4d shows the rank error curve of all algorithms, with MCount having better accuracy than others. Fig 4e shows the boxplot of both algorithms’ recognition error on each of the 960 sub-images, with MCount having a 3.99% average error rate, substantially lower than the average error rates of NICE / AutoCellSeg / OpenCFU of 16.54% / 33.54% / 50.31%.”

**[Comment 1-2]**

MCount has two major tunable hyperparameters. As I understood, this is not the case for NICE. I acknowledge the effort the authors have put into guiding users in how to determine those hyperparameters. Still, to understand to what extent the improved performance of MCount is due to those tuned hyperparameters, it would be interesting to see how MCount would perform 'out-of-the-box' without parameter tuning, and how this would compare to NICE. To assess this, the authors could test how optimized hyperparameters tuned on the current dataset perform on an independent dataset, ideally obtained in an independent setting (e.g. by another lab)

**[Response 1-2]**

We appreciate your concerns on hyperparameter tuning and would like to address them as follows.

**NICE Also Has Hyperparameters:** It's important to note that NICE, like MCount, includes hyperparameters, such as sigma, i.e. the threshold of Otsu algorithm used in NICE. While many users might default to the standard values, these parameters significantly impact NICE's performance. However, in practice, many biologists prioritize experimental efficiency or believe that the error introduced by hyperparameters is smaller than the inherent variability in biological experiments. As a result, the adjustment process in NICE often involves providing a single example image and manually tuning the parameters based on subjective judgment, and in many cases, researchers simply use the default values without further calibration.

**Necessity of Hyperparameters:** We believe that having tunable hyperparameters is essential for a tool like MCount, as it allows the tool to be more adaptable to different scenarios and achieve optimal performance. From a data science perspective, the best approach to obtain optimal hyperparameters is to construct a small, well-labeled dataset that reflects the typical experimental setting, which can then be used to calibrate values like sigma. This approach ensures that MCount can maintain high accuracy across various experimental conditions and image qualities.

However, the choice of hyperparameters presents a dilemma: on one hand, we want them to be sensitive enough to ensure adaptability across different experimental conditions; on the other hand, we want them to be less sensitive, making the tool easier to use for those who prefer simplicity. Given that MCount has the more advanced capability to differentiate overlapping colonies, its parameters are necessarily more sensitive compared to NICE.

**Performance on Different Datasets:** While we did not conduct experiments on a completely new and independent dataset, as our dataset, which consists of 10 groups of 96 images each, already exhibits sufficient variance. These 10 groups vary in terms of colony size, density, and even the setup used during image collection (e.g., variations in camera positioning, background lighting, etc.). This natural diversity in the dataset makes it suitable for evaluating the robustness of MCount's performance. We added corresponding clarification in the main text (lines 213-216), which reads as follows:

“… These images were derived from 10 sets of 96-well plates, each with slightly different experimental conditions performed on different dates, ensuring that the dataset contains a certain degree of variation. These variations include differences in colony size, density, and the setup used during image collection, such as camera positioning and background lighting.”

To address the reviewer's concerns, we applied the optimized hyperparameters to these 10 groups and assessed MCount's accuracy. Below is a summary of the results, which has now been added into Supplemental Information as S2 Table. These results demonstrate that MCount maintains strong performance across varied conditions within the original dataset, with an average error rate that remains significantly lower than NICE (16.54%). This suggests that the optimized hyperparameters are robust and generalizable across different experimental settings.

| **Dataset** | **Colony Number** | | **Recognition Error Rate (MCount)** | | **Recognition Error Rate (NICE)** | |
| --- | --- | --- | --- | --- | --- | --- |
|  | **Mean** | **Variance** | **Mean (%)** | **Median (%)** | **Mean (%)** | **Median (%)** |
| 1 | 58.79 | 85.24 | 5.10 | 4.55 | 18.13 | 18.52 |
| 2 | 20.64 | 53.21 | 1.82 | 0 | 11.84 | 10.91 |
| 3 | 48.01 | 42.07 | 4.38 | 3.45 | 19.20 | 17.95 |
| 4 | 15.51 | 28.59 | 3.55 | 0 | 11.95 | 9.52 |
| 5 | 9.38 | 9.89 | 7.87 | 0 | 14.62 | 11.11 |
| 6 | 34.60 | 43.73 | 3.48 | 2.90 | 22.85 | 22.40 |
| 7 | 22.33 | 21.97 | 2.49 | 0.00 | 11.34 | 11.11 |
| 8 | 56.69 | 122.76 | 5.39 | 4.92 | 23.17 | 22.86 |
| 9 | 38.28 | 57.64 | 2.67 | 2.53 | 23.25 | 24.03 |
| 10 | 15.19 | 23.26 | 3.18 | 0 | 9.53 | 9.09 |

**S2 Table. Performance of MCount using globally optimized hyperparameters (**$\boldsymbol{\lambda=}\mathbf{26}$ **and** $\boldsymbol{d=}\mathbf{0.5}$**) across 10 sub-datasets.** The table presents the average error rates for each sub-dataset when applying the global optimal hyperparameters. The results indicate that MCount maintains robust performance across varied experimental conditions, with error rates significantly lower than those achieved by NICE.

**[Comment 1-3]**

Along those lines, how sensitive is the optimum of the hyperparameters to various datasets? Especially datasets with similar colony density but obtained with a different imaging setup?

**[Response 1-3]**

The sensitivity of the optimal hyperparameters can indeed be characterized from two perspectives:

**Effect of Hyperparameter Adjustment on Results**: The first perspective is to observe how adjusting the hyperparameters affects the results. We previously addressed this and presented the result in S3 Fig, which illustrates how variations in hyperparameters impact the performance of MCount.

**Variation of Optimum Across Different Datasets:** The second perspective, as the reviewer suggested, concerns whether the optimum hyperparameters differ across datasets, particularly those with similar colony density but obtained using different imaging setups. To explore this, we calculated the optimal hyperparameters for each of the 10 sub-datasets in our study. Again, these 10 sets vary in terms of colony size, density, and the imaging setup. The results have now been summarized in S1 Table as follows:

| **Dataset** | **Colony Number** | | **Optimized Hyperparameters** | | **Recognition Error Rate, Mean (%)** |
| --- | --- | --- | --- | --- | --- |
|  | **Mean** | **Variance** | $\boldsymbol{d}$ | $\boldsymbol{\lambda}$ |  |
| 1 | 58.79 | 85.24 | 0.1~0.4 | 10 | 3.65 |
| 2 | 20.64 | 53.21 | 0.1~0.4 | 28 | 1.51 |
| 3 | 48.01 | 42.07 | 0.5 | 20 | 3.43 |
| 4 | 15.51 | 28.59 | 0.6 | 36 | 1.58 |
| 5 | 9.38 | 9.89 | 0.7 | 60 | 1.36 |
| 6 | 34.60 | 43.73 | 0.1~0.4 | 24 | 3.09 |
| 7 | 22.33 | 21.97 | 0.6 | 32 | 2.11 |
| 8 | 56.69 | 122.76 | 0.1~0.4 | 18 | 3.99 |
| 9 | 38.28 | 57.64 | 0.1~0.4 | 26 | 2.67 |
| 10 | 15.19 | 23.26 | 0.6 | 50 | 1.56 |

**S1 Table. Variation of optimal hyperparameters across 10 sub-datasets generated under different experimental conditions.** The table shows the mean and variance of colony number, optimal hyperparameter values, and the corresponding recognition error rate for each sub-dataset. The result demonstrates minimal variation in hyperparameters across sub-datasets and consistently lower error rates compared to the global settings (3.99%).

These results indicate that while there is some variation in the optimal hyperparameters across different sub-datasets, MCount maintains robust performance. The differences in optimal hyperparameters are likely influenced by factors such as colony size, density, and variations in the imaging setup (e.g., camera positioning, background lighting). This analysis provides insights into the robustness and adaptability of MCount across different experimental conditions.

Additionally, we observed a pattern where the value of the hyperparameter 𝑑 tends to increase when the mean colony number decreases (considering a consistent culture time, which results in larger colonies). This correlation suggests that 𝑑 is positively related to colony size, which aligns with our expectations.

It's also worth noting that the recognition error rates for these individually optimized sub-datasets are significantly lower than the error rate observed when using a single set of optimal parameters for all 10 datasets combined (4%). This further underscores the benefit of tuning hyperparameters to suit specific experimental conditions.

Our assessment of MCount across different datasets has now been added to the main text (lines 320-331), which reads as:

**“**To gain insights into the sensitivity of MCount to hyperparameters, we explored two key questions. First, we examined whether the optimal hyperparameters for each sub-dataset significantly differed from the global optimal parameters, noting that these 10 sub-datasets were generated under varying experimental conditions. The results, as shown in S1 Table, indicate that the variation in optimal hyperparameters across different sub-datasets is minimal, and their corresponding recognition error rates are all lower than the error rate observed when using the global optimal hyperparameters (3.99%). Additionally, we observed a pattern where the value of the hyperparameter $d$ tends to increase as the mean colony number decreases. When culture time is consistent, sparser colony density typically leads to larger colony size, suggesting that $d$ is positively related to colony size. The second question we explored was how well the globally determined optimal hyperparameters perform across different sub-datasets. The results, presented in S2 Table, show that the globally optimized hyperparameters applied to these 10 groups allow MCount to maintain robust performance across varied conditions, with average error rates that remain significantly lower than NICE. …**”**

**[Comment 1-4]**

To determine the hyperparameter lambda from unlabelled data, the authors present a strategy building on a equidispersion criteria. I did not understand, whether this criteria leads to a unique optimum for lambda? If not, what are the implications for this approach?

**[Response 1-4]**

As the reviewer rightly pointed out, the equidispersion criterion does not guarantee a unique solution for the lambda parameter. Theoretically, there could be multiple lambda values that satisfy this criterion. However, this does not render the equidispersion criterion ineffective; rather, it serves as a tool to identify a range of lambda values that are relatively reasonable. We have added corresponding clarifications to the main text (lines 467-473):

**“**Notably, the equidispersion criterion helps identify a range of reasonable lambda values, though it may not always yield a unique solution. In practice, we often observe that within a reasonable range—typically near the default values—a single optimal lambda value emerges. This is because variance is generally less sensitive to changes in lambda, while the mean is more responsive, aiding in narrowing down the solution. When multiple lambda values meet the criterion, they are applied to the colony counting process and manually inspected to determine the most accurate outcome, with clear indications when overestimation or underestimation occurs.**”**

**Practical Observations:** In our experimental experience with various datasets, we typically observe that within a reasonable range (usually around the default values of the parameters), we tend to obtain a single solution for lambda. This is because variance is relatively insensitive to changes in lambda, while the mean is more responsive to variations in lambda, helping to narrow down the solution.

**Handling Multiple Lambda Values**: In cases where multiple lambda values meet the equidispersion criterion, we can use these values to perform the colony counting and then manually inspect the results to determine which value provides the most reasonable outcomes. We have encountered situations where certain lambda values resulted in clearly overestimated or underestimated colony counts, making it evident which lambda should be chosen.

**[Comment 1-5]**

The authors apply their method using a fluorescent strain. Is fluorescence imaging required for the good performance?

**[Response 1-5]**

While fluorescence imaging can enhance performance, it is not strictly required. We have also successfully applied our method to non-fluorescent images in other, unpublished research. For instance, we used our approach to count colonies with different morphological characteristics in images containing mixtures of colonies with three different colors or sizes. These experiments demonstrate that our method is versatile and can effectively adapt to non-fluorescent imaging scenarios, provided the images are of sufficient quality to distinguish between colonies.

In this study, fluorescence imaging was primarily used to facilitate the manual annotation of the dataset. Fluorescence provides clear and distinct colony boundaries, making it easier to create accurate ground-truth labels, which are essential for training and validating the model.

We have added corresponding edits to the main text as follows (lines 209-212):

“While our method is versatile and can effectively adapt to non-fluorescent imaging scenarios, the use of fluorescence imaging primarily facilitated the manual annotation process by providing clear and distinct colony boundaries, enabling more accurate ground-truth labeling essential for training and validating the model.”

**Response to Reviewer 2**

**[Comment 2-1]**

This work presents a classical image analysis workflow for counting CFUs. The method is described in all necessary detail, code and data are available, and the results are quantitatively analyzed. The main limitation of this manuscript is that it lacks a thorough comparison to existing literature and state-of-the-art. Apart from some qualitative comparison in the first figure and a few plots comparing their work to NICE in Fig. 4, no such comparison is performed. The literature cited is mostly from computer vision applications to other types of overlapping objects, but not microbiology or bioimaging.
The problem of counting objects is nowadays usually solved with object detection and/or segmentation neural networks, some of which are specifically taylored towards separating overlapping convex objects such as cell nuclei (e.g. Stardist, Cellpose). In the field of colony counting, some classical and ML-based literature not cited here (just as examples) include:

<https://doi.org/10.2144%2F000112018>
<https://www.nature.com/articles/s41598-020-72979-4>
<https://link.springer.com/doi/10.1007/s10796-009-9149-0>
<http://arxiv.org/abs/2009.00926>
<https://doi.org/10.1364/OSAC.396603>
<https://doi.org/10.1016/j.tim.2021.01.006>
<https://vciba.springeropen.com/articles/10.1186/s42492-022-00122-3>

The literature is for example reviewed in

<https://doi.org/10.1016/j.tim.2021.01.006>
<https://link.springer.com/article/10.1007/s10462-021-10082-4>

It is not necessary to cite all of this work in the manuscript, but the authors are encouraged to take the effort and spend some time studying the current literature, and then add a discussion of their work with respect to the current state of the art, and ideally also some more quantitative comparison.

**[Response 2-1]**

We appreciate the reviewer for pointing out this limitation in our manuscript. We have taken the following steps to address the comment.

As per the reviewer’s suggestion, we have incorporated additional references to bioimaging and neural network (or deep learning) related literature in the manuscript. We also provided a paragraph discussing on why we chose not to adopt neural network (or deep learning) approaches. We would like to clarify that, broadly speaking, our method can also be classified as a machine learning (ML) method, which is why we avoid labeling other approaches as exclusively "ML methods." Perhaps it would be more appropriate to refer to our approach as a "classical method" and to the methods the reviewer mentioned as "neural network methods." Changes were made in throughout the Introduction of main text, more specifically at lines 79-87 as follows:

“In addition to classical object counting methods, neural networks have been applied across many fields due to their success in processing complex tasks (22,37,38). However, when applied to colony counting, these methods encounter significant challenges, primarily due to the lack of standardized, large-scale, high-quality datasets. Most existing datasets are small, often only a few hundred images, which are insufficient for training deep learning models with numerous parameters. Additionally, neural networks often function as "black boxes", which may provide final counts without visually delineating individual colonies, a drawback for biologists who need visual confirmation. While advanced models could include features like contouring, they require more robust datasets and computational resources. For these reasons, classical algorithms remain a more suitable choice for colony counting.”

We have made an effort to include more quantitative comparisons in our revised manuscript. Specifically, we have modified Figure 4 to further include the counting performance of OpenCFU and AutoCellSeg. We believe this comparison is reasonable because, as the reviewer noted, one of the 2022 ML papers referred also uses OpenCFU and AutoCellSeg as benchmarks. We also attempted to include comparisons with newer tools, such as the ones referred above. However, we encountered several challenges: some of these tools do not provide source code, making replication difficult; others offer software that we were unable to download, or that did not function correctly after download. The details of these challenges are as follows (papers are listed in the order of reviewer mentioned).

| **Analysis of Papers (Listed in Order of Mention by the Reviewer)** | | | | |
| --- | --- | --- | --- | --- |
|  | **Papers** | **Year** | **Method** | **Analysis and Notes** |
| 1 | <https://doi.org/10.2144%2F000112018> | 2005 | Classical | 1. This [software](http://www.cs.tut.fi/sgn/csb/cellc) is no longer available for download 2. This software is designed for cell counting rather than colony counting. 3. Given the algorithm used and the publication year, we anticipate that this software would perform worse than NICE, OpenCFU, or AutoCellSeg. 4. This paper has been cited as a reference. |
| 2 | <https://www.nature.com/articles/s41598-020-72979-4> | 2020 | Classical | 1. This [software](https://github.com/ColTapp) struggles with low-resolution images, which are often used in high-throughput 96-well setups. Even with its example images, it fails to recognize any colonies when the image resolution is lower than 900x900.      2. The paper uses classical techniques such as local adaptive thresholding, watershed segmentation, and Hough transform. 3. The software is primarily designed for analyzing a series of images from the same plate, making it less effective for single-image analysis. 4. This paper has been cited as a reference. |
| 3 | <https://link.springer.com/doi/10.1007/s10796-009-9149-0> | 2009 | Classical | 1. This paper cannot be reproduced as it does not provide any source code or software. 2. The paper presents algorithms for colony counting and classification, using classical techniques such as Otsu thresholding and watershed algorithms. Therefore, we anticipate that this software would perform worse than NICE, OpenCFU, or AutoCellSeg. 3. This paper has been cited as a reference. |
| 4 | <http://arxiv.org/abs/2009.00926> | 2020 | Neural Network | 1. This paper cannot be reproduced as it does not provide any source code or software, and the algorithm descriptions are vague. 2. The paper describes the application of neural networks to colony counting, but it is not well designed or written. Key issues include: (1) the dataset contains only 108 images, which is insufficient for evaluating colony counting; (2) the technical description is lacking, particularly regarding image preprocessing; (3) there is no detailed description of the network structure or parameters, which are essential in neural network papers; (4) the image quality is concerning, with inadequate descriptions provided. 3. Due to these concerns, this paper was not cited. |
| 5 | <https://doi.org/10.1364/OSAC.396603> | 2020 | Neural Network | 1. This paper cannot be reproduced as it does not provide any source code or software. 2. The algorithm described is designed for cell counting rather than colony counting, and its performance evaluation is based on cell images. 3. This paper has been cited as a reference. |
| 6 | <https://doi.org/10.1016/j.tim.2021.01.006> | 2021 | N/A | 1. This paper is a review paper. 2. The main focus is on algorithms for cell recognition rather than colony recognition. It discusses three papers related to colony counting, but none of them provides code or software for reproduction. 3. This paper has been cited as a reference. |
| 7 | <https://vciba.springeropen.com/articles/10.1186/s42492-022-00122-3> | 2022 | Classical | 1. This [software](https://github.com/louis8765/CFUCounter) could not be properly installed: (1) The authors note that it may not run on Windows, and indeed, we failed to install the Windows version. (2) We also failed to install the Mac version, as it seems to be compatible only with certain Mac systems      2. Despite using “machine learning” in the title, the algorithms employed are classical methods, including computer vision techniques such as watershed algorithms. Therefore, it is unclear how it could perform better than current benchmarks. 3. Notably, this paper uses OpenCFU and AutoCellSeg as benchmarks for performance evaluation. 4. This paper has been cited as a reference. |
| 8 | <https://doi.org/10.1016/j.tim.2021.01.006> | 2021 | N/A | This paper is the same as Paper 6 |
| 9 | <https://link.springer.com/article/10.1007/s10462-021-10082-4> | 2021 | N/A | 1. This review paper is well written has been cited as a reference. |

**[Comment 2-2]**

Some technical remarks: it would be great if more documentation, explanation of the individual steps, and installation instructions could be added to the Jupter Notebooks. The figures appear in very low quality in the PDF, but this might be a production issue unrelated to the submitted material.

**[Response 2-2]**

We have updated the Jupyter Notebooks by adding additional documentation and comments to clarify the individual steps. We have also renamed some variables and functions to make the code more intuitive. We hope these changes improve the clarity and usability of the notebooks. The updated Jupyter Notebook is publicly available on Dryad (<https://doi.org/10.5061/dryad.2280gb62f>).

Regarding the figure quality issue, we would like to clarify that the version we submitted to PLOS ONE included the text and images uploaded separately. The text was provided in a DOCX format, and the images were submitted as high-resolution TIFF vector files. We did not upload any PDF versions of the images. Therefore, we guess the low image quality the reviewer mentioned here may be a production issue that occurred during the review process.
